# Mortality Related to Acute Illness and Injury in Rural Uganda: Task Shifting to Improve Outcomes

**DOI:** 10.1371/journal.pone.0122559

**Published:** 2015-04-07

**Authors:** Stacey Chamberlain, Uwe Stolz, Bradley Dreifuss, Sara W. Nelson, Heather Hammerstedt, Jovita Andinda, Samuel Maling, Mark Bisanzo

**Affiliations:** 1 Global Emergency Care Collaborative, Massachusetts, United States of America and Uganda; 2 Department of Emergency Medicine and Center for Global Health, University of Illinois at Chicago, Chicago, Illinois, United States of America; 3 Department of Emergency Medicine, University of Arizona, Tucson, Arizona, United States of America; 4 Department of Emergency Medicine, Maine Medical Center, Portland, Maine, United States of America; 5 Idaho Emergency Physicians, Boise, Idaho, United States of America; 6 Karoli Lwanga Hospital, Nyakibale, Uganda; 7 Department of Psychiatry, Mbarara University of Science and Technology, Mbarara, Uganda; 8 Department of Emergency Medicine, University of Massachusetts, Worcester, Massachusetts, United States of America; International AIDS Vaccine Initiative, UNITED STATES

## Abstract

**Background:**

Due to the dual critical shortages of acute care *and* healthcare workers in resource-limited settings, many people suffer or die from conditions that could be easily treated if existing resources were used in a more timely and effective manner. In order to address this preventable morbidity and mortality, a novel emergency midlevel provider training program was developed in rural Uganda. This is the first study that assesses this unique application of a task-shifting model to acute care by evaluating the outcomes of 10,105 patients.

**Methods:**

Nurses participated in a two-year training program to become midlevel providers called Emergency Care Practitioners at a rural district hospital. This is a retrospective analysis of the Emergency Department’s quality assurance database, including three-day follow-up data. Case fatality rates (CFRs) are reported as the percentage of cases with a specific diagnosis that died within three days of their Emergency Department visit.

**Findings:**

Overall, three-day mortality was 2.0%. The most common diagnoses of patients who died were malaria (n=60), pneumonia (n=51), malnutrition (n=21), and trauma (n=18). Overall and under-five CFRs were as follows: malaria, 2.0% and 1.9%; pneumonia, 5.5% and 4.1%; and trauma, 1.2% and 1.6%. Malnutrition-related fatality (all cases <18 years old) was 6.5% overall and 6.8% for under-fives.

**Interpretation:**

This study describes the outcomes of emergency patients treated by midlevel providers in a resource-limited setting. Our fatality rates are lower than previously published regional rates. These findings suggest this model of task-shifting can be successfully applied to acute care in order to address the shortage of emergency care services in similar settings as part of an integrated approach to health systems strengthening.

## Introduction

Acute care needs are ubiquitous worldwide. Patients present with infections, dehydration, injuries, and acute exacerbations of chronic illnesses, regardless of the health care system’s current capacity to treat them. In resource-limited settings, these emergency conditions are amplified by a lack of primary healthcare and deficiencies in health infrastructure and public safety measures. As a result, the top causes of under-five mortality in sub-Saharan Africa (SSA) are malaria, diarrhea, and pneumonia, three conditions that are treatable with low cost interventions available widely in low-resource settings [1]. A significant factor contributing to poor patient outcomes in these settings is an overall paucity and inequitable distribution of health care workers [2]. In rural areas in particular, where child mortality rates are the highest, physicians are notably in short supply [3]. Thus, especially in rural areas, treatment of patients with acute illness and injuries falls on non-physician clinicians who are not trained to recognize emergencies and intervene in a timely fashion.

Given the severity of the human resource gap in many low-income countries, western models of emergency care development, focusing primarily on physician training, are not practical to address the pressing needs of patients in the next several decades. Emergency physician specialist training remains in its infancy or non-existent in many SSA countries, and most physician training is focused in urban areas [4, 5]. Even when there are significant cohorts of emergency physicians trained, which will take decades to accomplish, it is very unlikely that the rural emergency care needs will be met by the limited number of physician specialists. Limitations to specialty physician staffing include problems of “brain drain”, poor regional retention, and relative high costs associated with training and employing specialty physicians in lower volume settings. For a country such as Uganda, with more than 84% of its population living in rural settings, the dire need for emergency care will not be met solely by specialist physician training [6]. Alternative and novel strategies are needed to address this void in order to make emergency care accessible to all.

One such strategy involves task-shifting to train midlevel providers in emergency care. Task-shifting is defined by the World Health Organization (WHO) as “a process of delegation whereby tasks are moved, where appropriate, to less specialized health workers.” Task-shifting can utilize the *existing* health care workforce to improve care through a redistribution of work. The model has been demonstrated to be effective in the health care sector in resource-limited settings, particularly in addressing HIV/AIDS and obstetric care, and has been promoted by the WHO as a means of strengthening health systems and increasing health workforce capacity [7, 8]. However, its application to emergency care has not been well studied [9].

This retrospective analysis of an Emergency Department (ED) quality assurance database is the first study to describe the results of using midlevel providers as independent emergency practitioners in a rural area of a low- or middle- income country. Prior to the development of the Emergency Care Practitioner (ECP) program, a needs assessment was performed to describe the patient characteristics and provider skills needed to treat this patient population [10]. This study builds upon the previously published work, using a large cohort of patients to evaluate the outcomes of patients who were treated primarily by ECPs rather than physicians.

## Methods

The study site is a rural district hospital in the Rukungiri District of Uganda that houses a six-bed ED staffed by ECPs, who are non-physician clinicians that completed a two-year advanced training course in emergency care. The Ugandan emergency medical system, hospital characteristics, training and skill set of the ECPs are described elsewhere [11]. During the duration of the study, ECPs assumed independent care for all ED patients. Hospital-based Ugandan physicians were on call for consultations, if required, particularly for surgical emergencies.

The Karoli Lwanga (Nyakibale) Hospital ED maintains a prospectively collected quality assurance database of all ED patient visits, including 3-day follow-up data on clinical status. This study is a retrospective analysis of that database, including all consecutive patient visits presenting to the ED between July 22^nd^ 2010 and March 14^th^ 2012, after which time the data collection method changed. When the database was developed, three-day follow-up was chosen for multiple reasons. Other studies have shown that between 50 and 77% of pediatric hospital mortality occurs within the first 48 hours of admission [12, 13]. Additionally, due to logistical challenges, three-day follow-up was thought to have a higher capture rate, thus minimizing loss to follow-up for discharged patients. Finally, mortality after three days might be less reflective of outcomes related to acute care provided in an ED.

Data were abstracted by research assistants (RAs) from the patient chart once the treating ECP completed the patient’s care. The first cohort of ECPs began practicing independently in March 2010; the ED quality assurance database was instituted on July 22^nd^, 2010. The dates for this study were chosen based on the availability of data from the time that the quality assurance database was instituted until the method of data collection changed in March 2012. There were three small gaps in follow-up data collection during the study period, when no RA was available. These gaps include September 24^th^ to September 28^th^, 2010; January 17^th^ to February 3^rd^, 2011; and February 13^th^ to February 27^th^, 2011.

Data contained in the ED quality assurance database includes: demographic information, chief complaints, vital signs at presentation, laboratory testing, radiographs ordered, bedside ultrasounds performed, procedures performed, diagnoses, condition upon ED discharge, and disposition. Three-day follow-up data were collected in person for all inpatients and by structured telephone interview for patients discharged (from the ED directly or from the hospital before the three-day follow-up). If the patient could not be contacted on the initial call attempt, additional attempts were made daily until ten days had lapsed since the ED visit. If the patient could not be contacted by day ten, he/she was declared lost to follow-up. All patient data was de-identified prior to data analysis. This study was approved by the Mbarara University of Science and Technology Institutional Review Board, the Uganda National Council for Science and Technology, and University of Massachusetts Institutional Review Board (reference number HS 1405).

Descriptive data are reported as percentages with 95% confidence intervals (CIs). Case fatality rates (CFRs) are reported for all patients (primary analysis) and for patients with follow-up (sensitivity analysis), as a percentage of deaths along with 95% CIs. Fisher’s exact test was used to compare age and gender data for patients with and without follow-up. All deaths were included in CFR calculations, and diagnosis-specific CFRs are for all patients with a given diagnosis, regardless of other diagnoses. CIs were calculated for all proportions using the Wilson method for binomial data [14]. Stata v12.1 (StataCorp, College Station, TX, USA) was used for all analyses.

## Results

During the 20-month period from July 2010 to March 2012, data were collected on 10,105 patient visits that were recorded in the Karoli Lwanga “Nyakibale” Hospital Emergency Department database. Table 1 shows the demographic and clinical characteristics of patients. There were 54.8% male visits and 21.1% visits for children under five years old (under-fives). Procedures were performed in 56.4% of patients. Laboratory testing, radiographs, and ultrasounds were performed in 57.2%, 6.6% and 4.6% of patients, respectively. Of the 10,105 patients, four were dead on arrival to the hospital (0.04%), 45 died in the ED (0.5%), and 58 (0.6%) left against medical advice. Of the remaining patients who were treated, 3,786 (37.5%) were discharged home, 40 (0.4%) went directly to the operating theatre for emergency surgery, and 5,991 (59.3%) were admitted (59.7% admission rate overall). The most common diagnoses were malaria (29.6%), trauma (17.2%), and pneumonia (9.6%).

**Table 1:** Characteristics of Patients Evaluated in the Nyakibale Emergency Department.

Demographics	N	Percent (95% CI)
Total	10,105	100.0
Gender		
Male	5528	54.8 (53.5 to 55.5)
Female	4551	45.2 (43.9 to 45.9)
Age		
< 1 year	714	7.1 (6.6 to 7.6)
1–4 years	1414	14.1 (13.3 to 14.7)
5–17 years	1753	17.4 (16.6 to 18.1)
18 years and older	6175	61.4 (60.0 to 62.0)
**Diagnostic Testing Performed**		
Laboratory Testing	5783	57.2 (56.3 to 58.2)
Radiographs	668	6.6 (6.1 to 7.1)
Any Ultrasound (formal or bedside)	577	5.7 (5.3 to 6.2)
Bedside Ultrasounds*	467	4.6 (4.2 to 5.0)
**Procedures Performed**	5697	56.4 (55.4 to 57.3)
**Diagnoses**		
Malaria	2965	29.5 (28.5 to 30.2)
Trauma	1735	17.2 (16.4 to 17.9)
Pneumonia	969	9.6 (9.0 to 10.2)
Malnutrition	322	3.2 (2.9 to 3.5)
HIV	279	2.8 (2.5 to 3.1)
Dehydration	276	2.7 (2.4 to 3.1)
Tuberculosis	270	2.7 (2.4 to 3.0)
Sepsis	211	2.1 (1.8 to 2.4)
Gastroenteritis	200	2.0 (1.7 to 2.3)
Diarrhea	59	0.6 (0.5 to 0.8)
Dysentery	58	0.6 (0.4 to 0.7)
**Disposition**		
Dead on Arrival	4	0.04 (0.02 to 0.1)
Died in the ED	45	0.5 (0.3 to 0.6)
Left Against Medical Advice	58	0.6 (0.4 to 0.7)
Discharged Home	3786	37.5 (36.5 to 38.4)
Admitted Directly to Operating Theatre	40	0.4 (0.3 to 0.5)
Admitted to Inpatient Ward	5991	59.3 (58.3 to 60.2)
Other	181	1.8 (1.6 to 2.1)

*At the time of this study, emergency point of care ultrasound was part of the Emergency Care Curriculum; however, this aspect of the Curriculum has been expanded and refined and in present day, emergency ultrasound use is more prevalent.

Of all 10,105 patients evaluated, 59.8% of patients had a single diagnosis while 29.6% had two diagnoses that included one of the following: malaria, pneumonia, sepsis, diarrhea or dehydration, tuberculosis, HIV, or malnutrition. Only 9.3% of patients had three diagnoses, and 1.3% of patients had four or more diagnoses.

Follow-up data were available for 6,214 patient-visits (61.5%). Follow-up rates for discharged and admitted patients were 52.2% and 67.9%, respectively. There were 201 deaths (overall mortality of 2.0%). Excluding patients who were dead on arrival, 22.8% of deaths occurred in the ED and 76.1% of deaths were in those admitted to the hospital (S1 Fig.). Only one death occurred in a patient discharged home; that patient’s chief complaint was “catheter change”, and he was diagnosed with a urinary tract infection and benign prostatic hypertrophy.

Case-based fatality rates (CFRs) are shown in Table 2 for all patients (primary analysis) and for only patients with three-day follow-up data (sensitivity analysis), listed by the top nine ED diagnoses. Overall mortality was 2.0%, and for under-fives it was 2.7%. Diagnosis-related all-ages CFRs were 2.0% for malaria, 5.5% for pneumonia, 6.6% for sepsis, and 1.2% for trauma. CFRs for under-fives were 1.9% for malaria, 4.1% for pneumonia, 11.8% for sepsis, and 1.6% for trauma. CFRs for patients with follow-up data were higher than those for all patients since the latter included patients lost to follow-up (mostly discharged home) in the denominator.

**Table 2:** Case Fatality Rates for Nyakibale ED Patients by Diagnosis.

	Case Fatality for All Patients				Case Fatality for Patients with Follow-up			
	All Ages		< 5 Years		All Ages		< 5 Years	
Diagnosis	n/N	% (95% CI)	n/N	% (95% CI)	n/N	% (95% CI)	n/N	% (95% CI)
**Dysentery**	2/58	3.5 (1.0 to 11.7)	0/14	0.0 (0.0 to 21.5)	2/32	6.3 (1.7 to 20.2)	0/11	0.0 (0.0 to 25.9)
**Gastroenteritis**	6/200	3.0 (1.4 to 6.4)	2/70	2.9 (0.8 to 9.8)	6/139	4.3 (2.0 to 9.1)	2/42	4.8 (1.3 to 15.8)
**Sepsis**	14/211	6.6 (4.0 to 10.8)	8/68	11.8 (6.1 to 21.5)	14/153	9.2 (5.5 to 14.8)	8/53	15.1 (7.9 to 27.1)
**Tuberculosis**	18/270	6.7 (4.3 to 10.3)	1/6	16.7 (3.0 to 56.4)	18/212	8.5 (5.4 to 13.0)	1/4	25.0 (4.6 to 69.9)
**HIV/AIDS**	18/279	6.5 (4.1 to 10.0)	6/34	17.7 (8.4 to 33.5)	18/221	8.1 (5.2 to 12.5)	6/30	20.0 (9.5 to 37.3)
**Trauma**	20/1735	1.2 (0.8 to 1.8)	3/191	1.6 (0.5 to 4.5)	20/1055	1.9 (1.2 to 2.9)	3/122	2.5 (0.8 to 7.0)
**Malnutrition**	21/322	6.5 (4.3 to 9.8)	20/295	6.8 (4.4 to 10.2)	21/280	7.5 (5.0 to 11.2)	20/256	7.8 (5.1 to 11.8)
**Pneumonia**	53/969	5.5 (4.2 to 7.1)	21/517	4.1 (2.7 to 6.1)	53/698	7.6 (5.9 to 9.8)	21/348	6.0 (4.0 to 9.1)
**Malaria**	60/2965	2.0 (1.6 to 2.6)	18/962	1.9 (1.2 to 2.9)	60/1962	3.1 (2.4 to 3.9)	18/626	2.9 (1.8 to 4.5)
**Overall**	201/10105	2.0 (1.7 to 2.3)	58/2129	2.7 (2.1 to 3.5)	201/6214	3.2 (2.8 to 3.7)	58/1377	4.2 (3.3 to 5.4)

As shown in Table 3, the most common diagnoses recorded in deceased patients (n = 201) were malaria (29.9%), pneumonia (26.4%), malnutrition (10.4%), and trauma (10.0%). For patients with only a single diagnosis (n = 154), the top three causes of death were malaria (22.7%), trauma (12.3%), and pneumonia (11.7%). For the subset of patients who were dead on arrival or died in the ED, the three most common diagnoses recorded were pneumonia (24.5%), trauma (20.4%), and malaria (14.3%), with all other diagnoses each accounting for less than 5%.

**Table 3:** Diagnoses Associated with Deaths.

	All Deaths (N = 201)		Deaths with Single Diagnosis(N = 154)		Deaths with Multiple Diagnoses(N = 47)	
Diagnosis	n	% (95% CI)*	n	% (95% CI)**	n	% (95% CI)*
**Malaria**	60	29.9 (23.6 to 36.7)	35	22.7 (16.4 to 30.2)	25	53.2 (38.1 to 67.9)
**Pneumonia**	53	26.4 (20.4 to 33.0)	18	11.7 (7.1 to 17.8)	35	74.5 (59.7 to 86.1)
**Malnutrition**	21	10.4 (6.6 to 15.5)	8	5.2 (2.3 to 10.0)	13	31.7 (19.6 to 47.0)
**Trauma**	20	10.0 (6.2 to 14.9)	19	12.3 (7.6 to 18.6)	1	2.1 (0.1 to 11.3)
**Tuberculosis**	18	9.0 (5.4 to 13.8)	5	3.3 (1.1 to 7.4)	13	27.7 (15.6 to 42.6)
**HIV**	18	9.0 (5.4 to 13.8)	4	2.6 (0.7 to 6.5)	14	29.8 (17.3 to 44.9)
**Sepsis**	14	7.0 (3.9 to 11.4)	7	4.6 (1.8 to 9.1)	7	14.9 (6.2 to 28.3)
**Gastroenteritis**	6	3.0 (1.1 to 6.4)	3	2.0 (0.4 to 5.6)	3	6.4 (1.3 to 17.5)
**Dysentery**	2	1.0 (0.1 to 3.5)	1	0.7 (0.01 to 3.6)	1	2.1 (0 to 11.3)

*Percents do not add to 100% because of multiple diagnoses per case.

** Percents do not add to 100% because of other, not reported diagnoses.

A comparison of those with and without follow-up data, shown in Table 4, shows no significant difference for gender (p = 0.38) or adult versus pediatric age classification between the groups (p = 0.46). However, patients with follow-up data had a higher proportion of eight of the top nine diagnoses, with trauma being the exception.

**Table 4:** Comparison of Patient Visits with and without Follow-up.

	Patient Visits with Follow-up, % (95% CI)N = 6214	Patient Visits without Follow-up, % (95% CI)N = 3891
**Gender**		
Male	54.5% (53.2 to 55.7%)	55.5% (53.9 to 57.0%)
**Age**		
0–4 years	22.2% (21.2 to 23.2%)	19.5% (18.3 to 20.8%)
5–17 years	16.7% (15.8 to 17.7%)	18.6% (17.4 to 19.8%)
18 years and older	61.5% (59.9 to 62.3%)	61.9% (60.4 to 63.4%)
**Diagnoses**		
Malaria	31.6% (30.4 to 32.7%)	25.8% (24.5 to 27.2%)
Trauma	17.0% (16.1 to 18.0%)	17.4% (16.3 to 18.6%)
Pneumonia	11.2% (10.4 to 12.0%)	7.1% (6.3 to 7.9%)
Gastroenteritis/Dehydration	5.8% (5.2 to 6.4%)	3.5% (3.0 to 4.2%)
Malnutrition	4.5% (4.0 to 5.0%)	1.1% (0.8 to 1.5%)
HIV	3.5% (3.1 to 4.0%)	1.6% (1.2 to 2.0%)
Tuberculosis	3.4% (2.9 to 3.8%)	1.6% (1.2 to 2.0%)
Sepsis	2.5% (2.1 to 2.9%)	1.5% (1.2 to 1.9%)
**Disposition**		
Left Against Medical Advice	0.5% (0.4 to 0.7%)	0.7% (0.5 to 1.0%)
Admitted Directly to OR	0.5% (0.3 to 0.7%)	0.3% (0.1 to 0.5%)
Admitted	65.3% (64.2 to 66.5%)	49.7% (48.1 to 51.2%)
Discharged Home from ED	31.9% (30.7 to 33.1%)	46.3% (44.8 to 47.9%)

## Discussion

Acute and emergency care needs have been a neglected public health challenge in resource-limited settings, particularly in rural areas. The Emergency Care Practitioner program described here is a unique application of a task-shifting model to the acute care environment that helps meet this challenge. It is important to note that the construct of task-shifting requires a redistribution of tasks to be performed in a safe and effective manner. This study looks at patient outcomes for patients treated by ECPs to determine the safety and efficacy of this acute care model.

Prior to the inception of the ED and ECP Program at Nyakibale Hospital, the hospital functioned similarly to other district level hospitals in SSA, with no formal triage protocols and no dedicated resuscitation area for ill or injured patients. Due to staffing limitations and lack of emergency care training, the average wait time to see a physician for admitted patients was 26 hours (unpublished data, Bisanzo and Ilgen, 2007). As such, there were often significant delays for patients to get acute resuscitation or diagnostic testing and definitive treatment.

Since the institution of the ED and ECP program, the majority of patients requiring hospital admission and/or urgent procedures are evaluated in the ED (open daily from 8am until 10pm). ECPs independently assess, diagnose, stabilize and treat patients, and determine disposition. Additionally, ECPs write initial admitting orders for the patients’ ongoing care on the wards. All diagnostic tests and procedures (including emergency bedside ultrasound and lumbar punctures) as well as therapeutic procedures (including wound care, incision and drainage, simple and complex laceration repairs, splinting, open and closed fracture reductions, paracenteses, and procedural sedations) are performed by the ECPs.

Published data for disease-specific case fatality rates in Uganda and similar locales in SSA is scant. However, patient outcomes in this study compare favorably to what little data are available. Malaria is still the leading cause of disease burden in Uganda. It is estimated to account for 15 to 20 percent of all hospital admissions and 9 to 14 percent of all hospital deaths nationwide [15, 16]. Our study similarly found malaria to be the leading diagnosis in all patients and the leading diagnosis in patients who died. The malaria case-fatality rate in this study was 2.03% overall with 1.87% for the under 5 group. These rates are markedly better than the national malaria case fatality rate of 3%, especially given that the national statistic is a global estimate, including non-hospitalized patients [17]. In addition, a recent study from a hospital in Tanzania reported a 3.2% CFR, and the pediatric rate was 4.2% at a single-center study in Uganda and 4.3% in a multicenter study in SSA [12, 18, 19]. Using the Kendjo multicenter study as comparison for mortality in severe malaria in under-fives, our study would suggest the number needed to treat (NNT) with emergency care in order to prevent one death from pediatric malaria is 41.

Trauma is increasingly recognized as a major public health concern in SSA [20]. Road traffic injuries have climbed to the eighth leading cause of death and the tenth leading cause of disability in SSA overall, and the leading cause of death and disability in males from ages 20–24 [21]. In Uganda, road traffic injuries account for 11% of all morbidity and mortality, and a recent study in Kampala found that 25% of all deaths were due to injuries, versus 7% in the United States [22, 23]. CFRs for trauma range between 2.7 and 3.8% regionally [24, 25]. In our univariate analysis, for patients with a single diagnosis, trauma was the second leading cause of death, and CFRs of 1.2% overall and 1.6% for the under-fives compare favorably with these other reports. However, many of the published studies are in urban settings, and potential differences between urban trauma patients and this rural patient population (in trauma severity, socioeconomic status of patients, geographic factors, and transportation, among other factors) may limit this comparison.

Pneumonia was the third most common diagnosis found in ED patients in this study. The CFRs of 5.5% overall and 4.1% in the under-five group, also compare favorably to a regional rate reported at one hospital of 15.5% overall and an under-five rate of 15.5% in another study [26, 27]. Other recent literature reports under-five CFRs for pneumonia of 3.9–5.5% in a non-HIV subgroup and up to 25.9% in an HIV group of hospitalized patients [28, 29]. HIV testing was not routinely performed on patients in this study, so CFRs represent a mixture of HIV positive and negative patients.

Loss to follow-up (LTFU) has been a significant problem in many programs targeting populations in SSA. This has been best studied in antiretroviral therapy programs for patients with HIV/AIDS where 40% of patients were found to be LTFU in a systematic review, and nomograms have been developed to correct mortality rates for LTFU [30, 31]. However, these studies are based on patients with chronic illness with longer term follow-up of one to two years. There are no similar studies of LTFU for acute care patients. In our comparison of patient-visits with and without follow-up data, while demographic variables were not different, those with follow-up had higher rates of all of the top nine diagnoses (with the exclusion of trauma). This finding is likely due to a higher severity of illness in admitted patients, who accounted for a greater percentage of those with follow-up.

The diagnoses with the highest case fatality rates overall were tuberculosis, sepsis, malnutrition, and HIV/AIDS. These findings are important to demonstrate the additional challenges faced by EDs in low-resource settings, where hospitals most lacking in resources are the same hospitals treating patients with the highest burden of disease. This is particularly true for patient populations with high HIV prevalence and poor nutritional status. These unique challenges to EDs in low-resource settings make the need for improved acute care services for all even more compelling.

### Limitations

Follow-up data was only available for 61.3% of patients seen in the ED. Follow-up rates were better for admitted patients (67.6%) than discharged patients (52.1%). Almost half of the patients lacking follow-up data (45.7%) are those who were discharged from the ED and not admitted to the hospital, therefore likely representing patients with less severity of illness. As the nearest hospital to Nyakibale Hospital is three hours away by car, re-admission to another hospital is unlikely, but theoretically possible. Therefore, although there was no follow-up for these patients, they are presumed to have survived to three days, but there is no certainty of this. Given this limitation, we have provided CFRs for both patients with and without follow-up data. Based on our assumption that most patients lacking follow-up survived to three days, our primary analysis reports this cohort, but a secondary analysis provides a more conservative estimate of only those with follow-up data.

Follow-up data was for three-day follow-up. This time frame was chosen for multiple reasons cited in the Methods section. While the majority of hospital mortality is likely to have occurred during this time, there is a possibility that mortality rates might be underestimated if patients died after three days. Additionally, while the study looks at consecutive patients treated over a 20 month period, there were three small gaps in data collection comprising 38 days in total due to lack of RA availability. These occurred due to logistical issues coordinating RA presence in country and were not isolated days, but rather three short groups of days. All other patients presenting to the ED during the study period are included in this analysis.

Although this study aims to describe outcomes of patients treated by midlevel (ECP) providers in emergency care, throughout the study period, volunteer emergency physicians (primarily U.S.-trained) were variably present in the ED for the purposes of bedside and didactic teaching. These volunteers were to serve in a teaching role and instructed not to directly participate in patient care; however, this dataset does not allow us to determine what effect their teaching may have had on clinical decision-making by the ECPs and patient outcomes.

Finally, a significant limitation exists in interpretation of the data with limited published statistics available for comparison. Patient outcomes appear to be better than comparable resource-limited hospitals with a similar spectrum of disease, but the paucity of published data and potential variability in patient populations and regions limits the interpretation of a direct comparison of case-based fatality rates. The lack of baseline data at this site, common in resource-limited settings, precluded this comparison as well. If a model of emergency midlevel providers were to be replicated in other rural resource-limited settings, it would be useful to have baseline data at a single hospital site prior to the initiation of the program in order to more accurately evaluate program impact.

## Conclusions

A lack of emergency care services combined with a healthcare workforce gap in SSA prompted the novel application of a task-shifting model to train midlevel providers at this rural Ugandan Emergency Department. Mortality rates of patients treated at this hospital across the spectrum of disease are lower than other published rates. It appears from this data that non-physician clinicians with specialized training can provide effective emergency care in resource-limited settings. This model warrants further study as a feasible and cost-effective means of addressing this critical public health challenge in resource-limited settings.

## Supporting Information

S1 Dataset (XLTX)

S1 Fig Nyakibale ED Patient Visit Outcomes. (PDF)
