# Supplementary figures and images for: Mortality Related to Acute Illness and Injury in Rural Uganda: Task Shifting to Improve Outcomes
Source: PLoS One. 2015 Apr 7;10(4):e0122559. doi: 10.1371/journal.pone.0122559 (PMC4388510; doi:10.1371/journal.pone.0122559)

Figure 1 - Nyakibale ED Patient Visit Outcomes

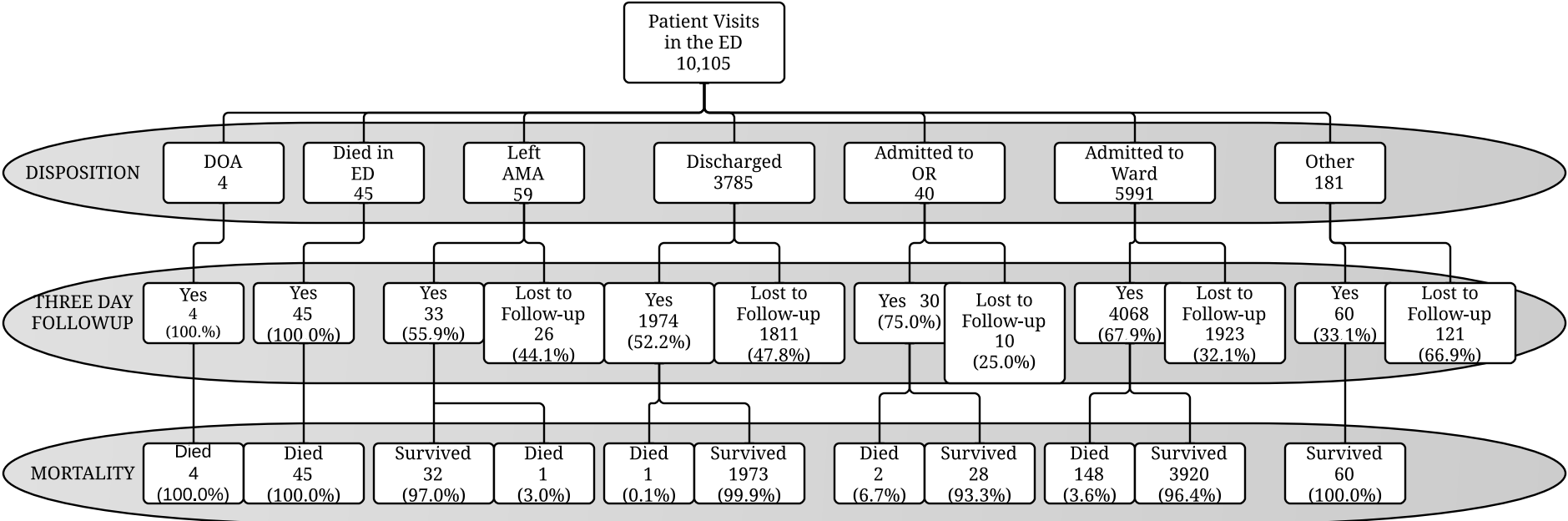

Supplement: S1 Fig — (PDF) [file pone.0122559.s002.pdf]
